# Lived Experiences of Returning to Participation After Mild Stroke: A Phenomenological Study in Spain

**DOI:** 10.1111/hex.70573

**Published:** 2026-02-24

**Authors:** Cristina de Diego‐Alonso, Almudena Buesa Estéllez, Javier Güeita‐Rodriguez, Pablo Bellosta‐López, Patricia Roldán‐Pérez

**Affiliations:** ^1^ Universidad San Jorge. Campus Universitario, Autov. A23 km 299, 50830, Villanueva de Gállego, MOTUS Research Group Zaragoza Spain; ^2^ Department of Physiotherapy, Occupational Therapy, Rehabilitation, and Physical Medicine, Research Group of Humanities and Qualitative Research in Health Science (Hum&QRinHS) Universidad Rey Juan Carlos Madrid Spain

**Keywords:** everyday life, life experience, participation, qualitative research, return, stroke

## Abstract

**Introduction:**

The process of returning to participation after a stroke depends on various individual and contextual factors, yet little is known about those with slight dependency. This study aimed to explore the experiences of people with mild sequelae after stroke in relation to their return to participation, considering the socio‐cultural context of Spain.

**Method:**

A qualitative phenomenological study was conducted involving 35 mild stroke survivors walking independently. The participants had experienced a stroke at least 6 months earlier. Data collection consisted of semi‐structured interviews and researchers' field notes. The analysis was conducted following Giorgi's method.

**Findings:**

Three themes were obtained: (a) Intrapersonal context: resilience to face sequelae and achieve autonomy, self‐reflections; (b) Return to daily life: home and leisure, work and study, daily community and travel; and (c) Interpersonal and social participation reengage: close connections, social bonds.

**Conclusion:**

Our findings suggest that returning to participation after a mild stroke is a highly heterogeneous process and is shaped by social support, adaptations facilitating independence, self‐determination, and the development of post‐stroke roles.

**Patient or Public Contribution:**

Individuals with lived experience engaged in the analysis and verification of the presented data. Their input helped ensure that the findings were interpreted in a way that reflects real‐world perspectives and relevance. No patient or public involvement occurred during the design or conduct of the study, or in the preparation of the manuscript.

## 1 Introduction

Stroke is the second leading cause of death and the third leading cause of disability worldwide [1]. It is a condition that requires prolonged treatment and accounts for approximately 34% of global health expenditures [2]. Globally, one in four individuals over the age of 25 will experience a stroke in their lifetime [1], resulting in limitations in daily activities and significant restrictions in participation [3, 4]. The broad concept of participation refers to the process of engaging in activities of daily life (e.g., household, social, leisure or travel) [5]. It involves a complex interaction among the person, the task and the environment, capturing what the individual can do, wants to do, and has the opportunity to do [6]. Participation also reflects the subjective experience of meaning, autonomy and self‐determination [7, 8]. For stroke survivors, participation in daily activities and community life [3], as well as previous roles [9], may take approximately 4 years [10].

A decline in participation increases dependency, affects family dynamics and leads to higher health and socio‐economic costs, consequences that could be mitigated through interventions aimed at promoting independence [2]. Previous studies have emphasised that an individual's health status, personal functioning level, access to services, and contextual and environmental factors all influence participation [11, 12, 13]. However, despite facilitating elements such as an extensive social network and support, the ability to drive, or independent ambulation, many stroke survivors still experience participation restrictions, particularly in social and leisure activities, employment and household tasks [3, 4, 10, 14, 15]. This limitation in actively returning to everyday life highlights the need for further exploratory research on the lived experiences of stroke survivors to better understand this situation. Qualitative data can help address gaps in outcome measures that primarily adopt a biomedical focus [10] and provide insight into the influence of additional factors. Specifically, understanding individual priorities and occupational history is essential to explain why stroke survivors maintain, discontinue or initiate long‐term activities [16]. Given the scarcity of research on the Spanish population [17], it is necessary to explore the perception of mild stroke survivors (i.e., no ambulation and communication impairment) after the first 6 months. This exploration aims to determine whether the experience of returning to participation among Spanish stroke survivors with mild impairment is comparable to that reported in other countries [18, 19, 20, 21, 22]. Previous research has shown that disability level can influence self‐care performance, while social participation may be affected by walking ability and unemployment [14]. Additionally, participation can be influenced by walking endurance and seasonal variability [23]. However, there remains a need to investigate unmet needs in individuals without severe sequelae more than 6 months post‐stroke [19].

Therefore, this study is the first to explore the experiences of individuals who have had a mild stroke regarding the process of returning to participation, along with conditioning factors (e.g., lifestyle outside the home, daylight hours and social activities) and the life circumstances of these individuals after the stroke, within the socio‐cultural context of Spain [24].

## 2 Materials and Methods

### 2.1 Procedure

The current study follows the guidelines of a qualitative research design [25] and is integrated into the multicentre Part&Sed‐Stroke project [26], which focuses on Spanish stroke survivors who are capable of walking and living independently, with no cognitive or communication impairments.

Employing a phenomenological approach [27] based on Husserl's framework [28], this research delves into understanding the lived experiences of individuals in specific circumstances [27], such as therapeutic interventions or health habits [28]. The primary goal of phenomenology is to explore phenomena as they appear, aiming to attain an essential comprehension of human experiences. Husserlian phenomenology is based on a relativistic ontology, from an interpretive epistemology in which knowledge is constructed and subjective. Lived experience is the way to grasp the essence of phenomena, through phenomenological reduction and eidetic description. The construction of data according to Giorgi's model is explicitly derived from these principles: it proposes the suspension of presuppositions (epoché), the descriptive analysis of units of meaning as they present themselves to consciousness, and eidetic synthesis to identify essential structures [29]. Both prior to and during the study, the researchers established their previous knowledge through two debriefing sessions, considering their beliefs and motivations for this research, thus ensuring reflexivity in the process [30].

### 2.2 Positionality Statement

Five researchers (three females) participated in this study, including two occupational therapists and three physiotherapists. Four are teaching and research staff at the university, with an average of over 10 years of clinical neurological experience, while the fifth is a full‐time research methodologist. None had clinical relationships with the participants at the time of the study. All researchers conducted the bracketing process at the beginning and throughout the research (Supporting Information I). This clinical experience in neurorehabilitation facilitated empathy and understanding of the context, but it could also generate assumptions that could be addressed through systematic bracketing, individual reflective reviews and contrast sessions between researchers. In addition to incorporating the initial and ongoing reflections of the principal investigator, the disciplinary diversity of the team allowed the team to identify blind spots, negotiate meanings and strengthen the credibility of the analysis.

### 2.3 Participants and Sampling Strategies

Participants for the qualitative study were sourced from the Part&Sed‐Stroke project [26], encompassing 140 individuals recruited from 13 specialised stroke rehabilitation centres across Spain. The inclusion criteria included stroke survivors over the age of 18 years, residing in their own homes, who were proficient in the use of technologies, with Functional Ambulation Categories ≥ 3. The exclusion criteria included cognitive impairment and communication alterations.

In phenomenological studies, it is common to include participants through purposive sampling based on specific purposes associated with addressing the research question or objective [27, 31]. The participants who met the inclusion criteria were recruited consecutively from the initial sample of subjects, and data collection ceased when the information obtained in the interviews became repetitive [31].

### 2.4 Data Collection

Data collection methods comprised both unstructured and semi‐structured interviews, augmented by researchers' field notes [31]. Initial data collection spanned from September 2021 to April 2022, evolving into a second phase from April 2022 to July 2022, marked by a triangulation process among the research team members to shape the semi‐structured interviews.

The first phase of data collection was conducted through unstructured interviews. In the second phase, a question guide was developed from the data obtained in the unstructured interviews held in the first phase (participants P01–P03) and through a triangulation process involving C.D.A., A.B.E. and P.R.P., which served as the basis for the format of the semi‐structured interviews (Table 1). In the second phase, semi‐structured interviews (Participants P04–P35) were used to obtain information on specific issues of interest based on the analysis of the responses from participants in the first phase.

**Table 1:** Interview guide.

Unstructured interviews
Can you tell me how having a stroke has influenced your participation in daily life activities?

Semi‐structured interview guide
What tasks do you no longer perform (both at home and outside) now?/ What activities do you avoid and why?/ What day‐to‐day tasks that are important to you inside and outside the home have you currently regained after the stroke?/ What factors prompt you or hold you back from resuming tasks after a stroke?/ What activities do you need or ask for help with, and what activities can you do on your own?/ What do you do now that you did not do before (new activities)?

All interviews were conducted and recorded after obtaining written consent from the participants. Each participant received a personalised email invitation. The Microsoft Teams platform (Microsoft, Redmond, the United States) was used, and recordings were transcribed verbatim, totalling 1194 min of interviews. Each participant was assigned an alphanumeric code to ensure confidentiality. The interviews were stored and password‐protected by the research team until analysis, and subsequently securely destroyed.

### 2.5 Data Analysis

During the data analysis stage, a model proposed by Amedeo Giorgi [32] was used. This method facilitated the identification of meaningful units, subsequent thematic categorisation and consensus‐driven theme finalisation.

**Figure 1:**
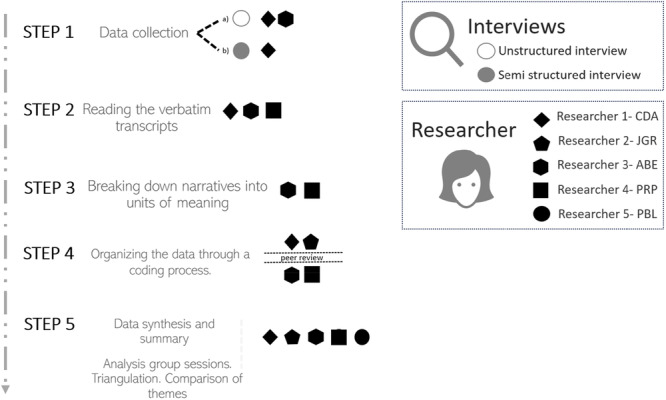
Description of the data analysis process.

In both interview phases, a coding grid was created with units of meaning, groups and identified themes. Relevant narratives were analysed, and data were compared and triangulated by two researchers [32]. Final themes were obtained through consensus among all researchers (Figure 1).

Notably, deviations from the published protocol included the absence of qualitative software for data analysis, and the Excel program was used instead, following a rigorous process [33]. This process consisted of systematically segmenting participants' narratives, assigning descriptive and thematic codes, and documenting analytic decisions through memos to ensure transparency and rigour.

### 2.6 Rigour and Quality Criteria

The Standards for Reporting Qualitative Research and the Consolidated Criteria for Reporting Qualitative Research were applied to accurately report on the study to ensure quality. The techniques and application procedures used to control reliability are described in Table 2 [34].

**Table 2:** Trustworthiness criteria.

Criteria	Techniques performed and application procedures
Credibility	Investigator triangulation: Team meetings were organised during the analysis of the situation, to design the questions for the semi‐structured interviews, to compare the results and to identify the final results. Some of the participants read the preliminary results and agreed with them.
Transferability	In‐depth description of the study, providing data and describing the study design and its different sections (context, research team, reflexivity process, sampling, inclusion criteria, data collection and analysis).
Dependability	Audit by an external investigator, responsible for the evaluation of the study protocol, with special attention to the method and implementation process during the study.
Confirmability	Researcher triangulation, triangulation of data collection. The reflexivity process was carried out by describing the positioning of the researchers and the reflexive debriefing by the researchers during data collection and analysis. An interpersonal reflexivity process was carried out during the different stages of the study.

### 2.7 Ethical Considerations

This study was approved by the Research Ethics Committee of the Aragon Community (internal registration number: PI21/333). Additionally, the study was conducted in accordance with the principles of the Declaration of Helsinki. The participants provided permission for audio and video recording through an informed consent form.

## 3 Findings

Thirty‐five stroke survivors participated in this study, residing in 24 different Spanish locations, including 23 men and 12 women aged 57 ± 12 years who had experienced a stroke 59 ± 44 months ago. Their mean Barthel Index score was 93 ± 9 (scale: 0–100), and the Functional Ambulation Categories score was 4.6 ± 0.6 (scale: 0–5). Regarding the level of participation measured with the Satisfaction with Daily Occupation‐Occupational Balance tool, they presented a total participation level of 7 ± 2 (scale: 1–13), and the total satisfaction level was 70 ± 12 (scale: 1–91). Of the 35 participants, only 6 lived alone and 12 required the support of a caregiver to perform daily life activities (Supporting Information II).

After coding and analysing the data from the 35 interviews, a total of 416 Units of Meaning (US) were identified. These were grouped and synthesised into three themes: ‘Intrapersonal context’, ‘Return to daily life’ and ‘Interpersonal and Social Participation Reengage’ (Supporting Information III, Table 3). Additional supporting quotes for each theme can be found in the Supporting Material IV.

**Table 3:** Themes, groups of common meaning and units of meaning identified during analysis.

Theme	Groups of common meaning	Units of meaning
Intrapersonal context	Resilience to face sequelae and achieve autonomy	
	Self‐reflections	
Return to daily life	Home and leisure	Previous activities already experienced
		New activities
	Work and study	Previously experienced roles
		New roles
	Daily commuting and travel	
Interpersonal and social participation reengage	Close connections	Family, couple and sexuality
	Social bonds	Performing in social life
		Engagement in healthcare

### 3.1 Intrapersonal Context

Participants shared their lived experiences of coping with the aftermath of stroke and the adaptation process they underwent. They highlighted the strength required to persist and achieve their goals, whether through sustained effort or small adaptations that facilitate participation in their environment. Finally, they reflected on how these experiences shaped their self‐perception. Although stroke is a condition no one would wish to experience, it can serve as a profound source of learning.

#### 3.1.1 Resilience to Face Sequelae and Achieve Autonomy

One of the most important aspects for patients inside has been that they have had to face the aftermath of the stroke. This confrontation has revealed annoying sequelae such as urinary incontinence, but it also shows the process of rebuilding oneself through resilience, going through what others think, the barriers of the environment and the effort to face problems and create novel solutions.

…the whole left side, I have no sensation … going to the bathroom, the sensation of “am I urinating or not?” Bowel movements, that sensation of “I need to go,” no, you don't identify the sensation very much.

P23

People care a lot about the physical aspect, don't they? So, you go to rehab (…): the emotional issues and what you feel and all that … they don't give any importance to that.

P02

I remember having trouble, for example, cutting meat. You go to a restaurant, they bring out a steak and a serrated knife, right? And then I would ask for the potato peeler. In the end, I bought a pocketknife.

P25

#### 3.1.2 Self‐Reflections

The participants extracted the essence of the experience through a brief conclusion, whether it was negative or positive.

I wouldn't wish this on my worst enemy.

P01

Well, I think it gives you an ability … to understand life and to know how strong you can get through the ups and downs that life throws at you. It's a, like a life experience.

P24

### 3.2 Return to Daily Life

This theme is divided into three sub‐themes that cover experiences related to participation in the environment, either in the dimension of home and leisure, as well as more intellectual aspects, such as work and study. Likewise, it deals with ideas related to mobility or being able to get around independently from one place to another.

#### 3.2.1 Home and Leisure

At home, as in leisure, participants encountered situations already familiar to them. When faced with these circumstances, two outcomes were observed: certain daily activities proved difficult or impossible to perform, while others continued to be carried out as before. This highlights the sense of well‐being derived from maintaining active participation in household tasks, even when assistance is required for some of them. Moreover, particularly in relation to leisure, the increased availability of free time following the stroke encouraged engagement in new activities, especially those connected to art.

Previous activities already experienced. At home, certain tasks previously performed by participants are now difficult or impossible to complete, prompting them to explain the reasons and whether they have chosen to delegate these tasks to others.

I can't make the bed; I can't mop either because I don't have enough balance…. Sometimes I do the dishes…

P06

Well, I used to do—everything that was home improvement—I did it myself. And now, of course, I don't do all this anymore for fear of not controlling my hand, right?

P11

I manage almost everything in the house; the children help me a little bit, but I am always the one who manages everything and that makes me feel good.

P21

New activities: This aspect refers to those activities that are practised now either because there is more time available than before or activities that were not practised or thought of before the stroke but are nevertheless being tried now.

Now I paint watercolours, with my left hand. I always liked to paint, but due to lack of time, I never dedicated time to it.

P02

I have a colleague who likes music a lot and sings, (…), he called me and I go and sing with him, and my wife says to me: “Hey, you would never have done this before.”

P34

#### 3.2.2 Work and Study

Understanding roles based on the tasks or functions each person performs allows for discussion of both previously experienced roles and newly developed functions.

Previously experienced roles: This previously experienced role could be related to study and work, and the role that the person with the sequelae of stroke now has changed. Even going back to work (recovering the role of worker) has become a utopia.

I gave up the student role because of the online classes, at the beginning it was hard for me to concentrate and then I didn't follow the teacher.

P13

At the beginning, the first few times I was put in as a senior resident (at the hospital) so I wouldn't be alone in the practice.

P34

I live my life the same as before. Well, related to work, I used to work and now I don't. That's what I miss: working.

P25

New roles. Participants reported taking on new activities or roles due to personal circumstances, most of which were closely related to household tasks. Moreover, these activities were performed in a different manner than before, without hurry or stress.

Now I have to cook more because I have a daughter, and I have to make menus and so on.

P22

It's true that I do things but it's another rhythm, it's another story. There is no stress, no tension, no responsibility, no hurry.

P19

#### 3.2.3 Daily Commuting and Travel

The freedom and independence to go from one place to another is an aspect that can determine a person's participation in the environment, affecting society, family, work or leisure areas.

Second point of independence. Very important: my adapted car. Of course, I drive an adapted car.

P22

However, going outside is often hindered by environmental factors. Crowded spaces increase feelings of insecurity and elevate the risk of falls.

Well, before the stroke I was very streetwise (…). After the stroke, my activity is at home, in the neighbourhood. Going out downtown is very difficult for me, because it is when there are lots of people…. I am afraid that someone will push me, and I will fall on the ground.

P13

### 3.3 Interpersonal and Social Participation Reengage

The participants explored distinct aspects of their interpersonal relationships in their living environment. The partner dimension was expressed as a strong support for the return to participation. That is, all those people in the close circle who represent an important support, be it the couple, family, close friends or support groups. However, social relationships or those in the healthcare setting were more variable. Some participants reflected on a reduced social life, while others expressed that their social relationships had increased after the stroke.

#### 3.3.1 Close Connections

The closest relationships are with the family and the couple. Although role changes within the household have already been discussed, this section focuses on the partner's role in overcoming or failing to overcome significant challenges. Participants highlighted aspects such as couple coexistence and joint strategies to address problems. They also discussed the importance of the sexual dimension, noting issues related to lack of knowledge, mobility limitations, fears and medication side effects.

I'm still a father, however my daughters pay the price, they have a broken father, because I am broken. And then, I know that there are things I can do, I mean, for example, I can do their homework with them.

P26

My father said that they were going to make me a room downstairs in the living room so I wouldn't have to climb stairs. And my girlfriend said, “no way,” that I was going to go up to the third floor (…) And, boy, did I manage….

P34

I haven't heard much about sexuality after stroke. I haven't recovered from it. It's a pending task that is there. I think it scares me a little bit, still.

P17

#### 3.3.2 Social Bonds

In this sub‐theme, we group the narratives of social bonds such as friendships and support groups. In relation to the latter, the link that patients have with the healthcare environment has also changed, and trust is valued more than anything else.

Performing in social life. The spectrum of experiences in this dimension is ambiguous. Some participants expressed that their social life had waned, whereas others perceived that after the stroke, their social life had recovered or even increased considerably. Some participants reflected that their social life had decreased in the dimension of interpersonal relationships, both face‐to‐face and through social networks.

Well, “my social life” … has changed. Maybe the mobility is not the same, but I still have a social life that is somewhat diminished, but hey, I have it.

P09

Likewise, the importance of support groups was highlighted, given that they generate a space for sharing among people in the same life situation.

So, this group of people who have had a stroke like me, the truth is that it helps us a lot and we are very happy to have it because they are great friends (…). And it's a very important support for us and for the setbacks we have.

P22

Some participants reported that they were gradually regaining their social life through participating in meaningful activities that motivated them to establish interpersonal relationships.

I am recovering,… the other day I went to a founding session of a new political party,… which was 13 h away from home (…) Well, it was a great effort, but well, it motivates me to recover my social life.

P18

Other participants indicated that their social life had improved after the stroke, either because they were not limited to being at home or because their new condition had made it easier to meet new people.

I don't go out because, of course, going out in a wheelchair is hard. And people are going to ask you. So, I wanted to stay at home, quietly, but I said to myself: “I'm not going to spend the whole summer at home. No, I'm not. I'm going out.” And then, on the contrary, afterwards I didn't want to go back home.

P02

Engagement in healthcare. The most highly valued relationships in the healthcare environment were those based on individualised treatment, trust and care and were more common in the context of associations.

…they have helped me a lot (the association), because … they give you confidence, the confidence to say that you can achieve, eh? And another thing: the warmth they have shown for each one of us, not only for me, but for everyone, the affection of all the people that help us.

P10

## 4 Discussion

The present study explored the experience of returning to participation after having had a stroke over 6 months ago. Thirty‐five people from various locations in Spain, living in their own homes and whose mild stroke sequelae did not limit their ability to be mobile, communicate or cognitively process, described the personal and social factors that have conditioned this experience. Experiences reported by a higher proportion of men reflect real‐life situations, as men tend to report greater independence after stroke [1]. These findings are particularly noteworthy because they capture the subjective experiences of individuals who, despite demonstrating high levels of independence (mean Barthel Index score of 93 out of 100 and Functional Ambulation Categories score of 4.6 out of 5), continue to face invisible conditions and a high prevalence of long‐term unmet needs [19, 35].

Our participants described their own experience after a stroke and the impact of its sequels, which, although mild, generated an impact on their daily lives. These findings highlighted the emotional context of the person, where the resistance to resume active participation coexisted with the intrinsic struggle to return to normality. This process was mainly mediated by the ability to accept and adapt, regardless of the detectable or invisible stroke sequelae, as other studies have shown [35, 36]. Therefore, emotional identification is pertinent [37] because the intrinsic motivation and post‐stroke attitude had a clear impact on persistence in resuming participation [11], with the person's occupational history [16] being relevant. These experiences align with self‐determination theory [8] and social cognitive theory [38], where motivation and experimentation strengthen self‐efficacy. The findings in our study extend the limited knowledge on coping in the chronic phase of stroke, highlighting the need for internal and social resources [39, 40]. Furthermore, it showed that only subjective experience provides insight into the conditions and strategies that support meaningful participation. However, more information is needed for people with mild injuries after several years [16, 41], often mistakenly considered as not needing adaptations [42]. In addition, the identity updating and adaptive behaviours identified are in line with current models, favouring realistic goals linked to improved well‐being and quality of life [16, 41]. The self‐reflection process on the lived experience provided them with a vital learning lesson that redefined their self‐concept. These findings are consistent with previous studies [42], in which each participant has reconstructed a new self through lived experiences in everyday life to consider their competencies, especially those that are meaningful to each person, and self‐efficacy prevails over functional level. Future studies are recommended to further explore self‐learning, especially metacognitive and self‐management components [39].

Participants described their current participation, comparing it to their pre‐stroke lifestyle and their current scale of priorities. In this way, the need to deepen the occupational history [5] of the stroke survivor and the identification of meaningful activities [43] is supported. These meaningful activities were found to be favourable for adherence and return to social participation and reintegration, contributing to quality of life and resilience [36, 44]. Furthermore, the results showed that the person makes an estimation of their abilities and limitations to perform autonomous participation, with special emphasis on their ability to adapt, search for resources and start unexplored activities as reported by stroke survivors after more than 15 years and their relatives in a study [16]. The study participants did not present severe restrictions in participation, which may be due to their mild stroke, as opposed to those with more severe sequelae who present restrictions in participation even after 5 years of age [45]. It could also be due to the self‐efficacy, which some showed identified as relevant to higher performance and satisfaction [7].

These results showed that the time elapsed since the injury is secondary to the participation return as compared to a multifactorial influence, as shown in previous studies [13, 16]. Therefore, although the passing of time influences the progressive functional recovery, it does not always imply a return to complete participation [10]. Stroke represents a turning point in people's lives [21], requiring a continuous and dynamic adjustment of personal identity and occupational roles [46], a process that never truly ends [21, 44, 47]. The individual's life situation must be considered, as environmental factors significantly influence opportunities to resume participation [20]. Engagement in activities reshapes and redefines roles, whereas roles simultaneously guide and sustain participation [5].

The main topics discussed by the participants were activities conducted at home and leisure, work and study, and the ability to get around. These may or may not be priority objectives in the rehabilitation plan [40], being necessary to adapt to the person's needs and demands [17, 48] due to their high potential for adherence in the process of returning to participation [12, 13, 36]. Household activities were often described in relation to the need or concern to seek and implement adaptations that support autonomy and independence. Leisure activities emerged as particularly meaningful, as participants reported having more time to enjoy, explore and resume activities that contribute to their sense of well‐being. Previous studies have contemplated similar aspects, referring to it as ‘reconstruction of the self’ [16]. Furthermore, evidence exists between the reciprocal effects of participation and well‐being after a stroke [41]. Participants indicated that their previously assumed roles had been disrupted. The results of our study reflected the need to resume these roles, either through adaptations or at a different pace [49], in order to cope with difficulties in performing simultaneous tasks, as well as with fatigue and physical limitations [50]. Moreover, our results revealed how stroke had generated an individual impact on the roles played so far, an unnoticed point in regard to the restructuring of the person after the stroke [16]. The ability to get around has been evaluated by previous studies [10]. Particularly, having a driver's license with some adaptations or not having to depend on others is in line with previous studies [48]. Specifically, a sense of independence in participating in social activities that is limited at times by fear of falling [51]. Although walking function is a key determinant of community mobility decline in chronic stroke, when this ability is preserved, other factors, such as those identified in this study, also influence mobility and warrant further investigation [52].

Regarding interpersonal relationships, our findings are in line with the literature highlighting the importance of the family [53]. However, our findings did not specifically mention the caregiver [54] even though 12 of the participants needed it, but the need for close help. Moreover, participants reflected the restrictions they encountered in their sexual relationship with their partner, information that was scarcely developed in previous studies and rarely included in intervention plans [55, 56], although it is in clinical guidelines [40]. Furthermore, interpersonal relationships in society are known to be a great support and facilitator of the return to participation after a stroke [48, 57], but the present study reported fluctuations in social life, at times increased or decreased after stroke. Participants looked for alternatives outside the family circle through friendship or support groups, placing value on finding connection points, which became crucial for adherence to participation [11]. External resources, such as healthcare and social support, play an important role in shaping individual and social resilience, yet they have received limited attention in the literature [36, 44]. Previous studies have highlighted the importance of group belonging and identification as key elements of psychological coping [58]. This aligns with the experiences reported by participants in our study, who described how these external resources influenced their process of re‐engagement.

Finally, participants expressed their experiences in relation to the different healthcare institutions with which they have come directly in contact, especially appreciating the individuality and closeness, but unfortunately, only supporting their potential in the new models of person‐centred healthcare [17, 59]. This study showed that, even when high levels of function and independence are maintained after stroke, individuals still undergo a significant process of adaptation to their new circumstances. These findings open an important debate on the need for rehabilitation services to also address the needs of people with mild stroke, particularly regarding social activity and participation [60].

### 4.1 Recommendations for Practice

This was the first study to describe the experience of participation after a stroke in people with mild sequelae, unrestricted walking and communication in different regions of Spain. Highly relevant aspects for clinical practice are collected, including the need to consider not only the capabilities and limitations of stroke survivors. But also, individual aspects regarding their self‐concept and self‐efficacy, needs, and interests, as well as searching for adaptations and scaffolding. Together, these foster independence and enjoyment, as these are the great potential for establishing adherence to active participation. Therefore, at the clinical level, it is suggested that the use of a qualitative interview may provide the most suitable way to understand these aspects hidden from quantitative assessments before setting goals and intervention plans focused on increasing participation. It is recommended to consider the life experiences of stroke survivors when co‐designing strategies for adaptation and reintegration into new participation contexts after a stroke, even in mild cases [60]. Empowering stroke survivors to actively seek resources from a self‐management perspective is essential [15, 23, 37, 45, 46].

### 4.2 Need for Further Research

Future studies should analyse the experience of the process of return to participation in people with mild sequelae during the first 6 months after stroke, to learn how they experience the process of change and accompaniment from the hospital healthcare environment to their home. We should also address the return to participation in people who are more dependent during the first 6 months as well as in the months and years after a stroke.

### 4.3 Strengths and Limitations

A strength of this qualitative study was the inclusion of 35 participants, a comparatively large sample within a population segment that is rarely examined [10, 12, 13]. This group is often overlooked because they are assumed not to experience difficulties in returning to participation [4] due to their high functional level and average age below 65 years [1]. In addition, this sample offered insights into a wide range of situations shaped by diverse socio‐cultural contexts, as participants were recruited from multiple regions across Spain. For these reasons, the results of this study highlight important aspects of the return‐to‐participation process that have been largely overlooked in previous research [10], may contribute to a better understanding of individual recovery trajectories and address the current gap in knowledge on this topic. This study provides new perspectives on the long‐term needs of people with mild stroke, a population in which the prevalence and heterogeneity of unmet needs remain high [19, 35]. The experiences and strategies reported by participants across different socio‐cultural and geographical settings in Spain [24] complement existing qualitative evidence on social participation and reintegration after stroke [11, 61].

However, the main limitation of this study is that these results cannot be extrapolated to all stroke survivors due to the nature of the research question, the selected qualitative design and the characteristics of the sample, which included only stroke survivors who were able to walk and had no communication impairment 6 months after stroke, with a wide variation in the time elapsed since stroke. Another limitation, despite employing a systematic process to minimise coding risks [33], was that the dataset was coded using Excel rather than computer‐assisted qualitative data analysis software such as NVivo, which could have reduced the potential risk of overlooking relevant elements within the narratives [62].

## 5 Conclusions

In conclusion, the results of this first study carried out with mild stroke survivors in Spain showed that the experience of participating 6 months after having had a stroke is an individual one and a high diversity of situations can be found, in spite of being able to move around and communicate, emphasising the importance of adaptations and social support to achieve independence, as well as the modification of roles and self‐determination after the situation experienced.

## Author Contributions

**Cristina de Diego‐Alonso:** data collection, triangulation process that served as the basis for the format of the semi‐structured interviews, semi‐structured interviews to obtain information on specific issues of interest based on the analysis of the responses from participants in the first phase, organising the data through a coding process, data synthesis, manuscript writing, reviewed the manuscript based on the comments provided in the decision letter, reviewed the final version after Revision 1, study conception or design, read and approved the final manuscript. **Almudena Buesa Estéllez:** data collection, triangulation process that served as the basis for the format of the semi‐structured interviews, breaking down narratives into units of meaning, organising the data through a coding process, data synthesis, manuscript writing, reviewed the manuscript based on the comments provided in the decision letter, reviewed the final version after Revision 1, study conception or design, read and approved the final manuscript. **Javier Güeita‐Rodriguez:** organising the data through a coding process, data synthesis, manuscript writing, reviewed the final version after Revision 1, study conception or design, read and approved the final manuscript. **Pablo Bellosta‐López:** data synthesis, manuscript writing, reviewed the final version after Revision 1, study conception or design, read and approved the final manuscript. **Patricia Roldán‐Pérez:** triangulation process that served as the basis for the format of the semi‐structured interviews, breaking down narratives into units of meaning, organising the data through a coding process, data synthesis, manuscript writing, reviewed the manuscript based on the comments provided in the decision letter, reviewed the final version after Revision 1, study conception or design, read and approved the final manuscript.

## Ethics Statement

This study was approved by the Research Ethics Committee of the Aragon Community (internal registration number: PI21/333). Additionally, the study was conducted in accordance with the principles of the Declaration of Helsinki.

## Consent

The participants provided permission for audio and video recording through an informed consent form.

## Conflicts of Interest

The authors declare no conflicts of interest.

## Supporting information


**Supplementary Material I:** BRACKETING (Positioning of researchers).

**Supplementary Material II:** Sociodemographic and clinical data of the participants.

**Supplementary Material III:** Themes, groups of common meaning and meaning units that emerged from the participants' narratives.

**Supplementary Material IV:** Additional supporting quotes for each theme.

## Data Availability

The data that support the findings of this study are available in the supporting material of this article.
