# Supplementary material for: Lived Experiences of Returning to Participation After Mild Stroke: A Phenomenological Study in Spain
Source: Health Expect. 2026 Feb 24;29(2):e70573. doi: 10.1111/hex.70573 (PMC12932911; doi:10.1111/hex.70573)
Supplement: Supplementary file 1 — Supplementary Material I: BRACKETING (Positioning of researchers). [file HEX-29-e70573-s001.docx]

**Supplementary material I.** BRACKETING (Positioning of researchers)

| **Previous theoretical framework** | Previously, the researchers had some background knowledge on the field derived from clinical experience and research consulted. |
| --- | --- |
| **What do you want to research?** | To explore the experiences of people who have had a stroke in relation to the process of returning to participation and the conditioning factors in the sociocultural context of Spain. |
| **Key informants** | Participants in the qualitative study were drawn from 13 specialised stroke rehabilitation centres across Spain. The informants were stroke survivors over 18 years of age, residing in their own home, able to use technology, able to walk independently and without cognitive or communication sequelae that limited the interviews. |
| **Beliefs regarding the lived experience** | Each person who survived a stroke experiences the process differently and there are various factors which influence their recovery process and directly influence the clinical rehabilitation intervention from occupational therapy and physiotherapy (especially in the family environment and the person's attitude).  Currently, rehabilitation models give little consideration to these factors and after 6 months focus on ‘maintaining the person’ without exploring options for change.  Approaches focusing on sensorimotor function do not have an impact on the person's day-to-day needs. Health education where the person is the main driver is not widely included.  The way of coping with the stroke situation is very different according to gender, especially male stroke survivors take on the role of patient despite increasing their functional level, but female stroke survivors resume their roles more quickly and care is usually provided by a hired caregiver and rarely by the husband. |
| **Previous experiences** | - Profession: Occupational therapist and physiotherapist specialising in the rehabilitation of people with adult neurological pathologies. Research teaching staff on the Degree in Physiotherapy and lecturer on postgraduate courses.  - Previous professional experience: professional experience as an occupational therapist and physiotherapist in the clinical care of people with neurological pathology in several public and private centres and associations. For 15 years she has provided care to different stroke survivors in different stages after the stroke (from the first months to several years after the stroke) and in different environments (hospital admission, outpatient and home care). The vast majority of the rehabilitation sessions have lasted about 60 minutes for several days a week over several months and even years, during which a strong bond has been established with some stroke survivors, going beyond the care setting and creating a space for trusting dialogue to address more personal aspects. |
| **Motivation to develop this research** | To make known the stories of people who have survived a stroke, what they need to increase their independence and well-being. |
