# Supplementary material for: Lived Experiences of Returning to Participation After Mild Stroke: A Phenomenological Study in Spain
Source: Health Expect. 2026 Feb 24;29(2):e70573. doi: 10.1111/hex.70573 (PMC12932911; doi:10.1111/hex.70573)
Supplement: Supplementary file 2 — Supplementary Material II: Sociodemographic and clinical data of the participants. [file HEX-29-e70573-s004.docx]

**Supplementary material II.** Sociodemographic and clinical data of the participants.

| **CODE** | **AGE (Years)** | **SEX** | **WORK STATUS** | **LIVING ARRANGEMENTS** | **CAREGIVER** | **MONTHS SINCE STROKE** | **BARTHEL INDEX**  **(100 max score)** | **FUNCTIONAL AMBULATION CATEGORIES** | **DAILY OCCUPATIONS-OCCUPATIONAL BALANCE**  **(Summed activity satisfaction)** | **DAILY OCCUPATIONS-OCCUPATIONAL BALANCE _**  **(Summed activity participation)** |
| --- | --- | --- | --- | --- | --- | --- | --- | --- | --- | --- |
| P01 | 58 | Woman | Disability pension | Lives with others | No | 139,2 | 70 | 5 | 9 | 89 |
| P02 | 50 | Woman | Disability pension | Lives with others | No | 114 | 90 | 4 | 10 | 80 |
| P03 | 54 | Woman | Disability pension | Lives with others | No | 34,8 | 75 | 4 | 8 | 43 |
| P04 | 62 | Men | Disability pension | Alone | No | 30 | 100 | 5 | 9 | 55 |
| P05 | 59 | Woman | Unemployed | Lives with others | No | 43,2 | 95 | 5 | 6 | 61 |
| P06 | 61 | Woman | Disability pension | Alone | No | 16,8 | 95 | 3 | 3 | 69 |
| P07 | 50 | Woman | Disability pension | Lives with others | Yes | 67,2 | 95 | 4 | 5 | 67 |
| P08 | 50 | Men | Disability pension | Alone | Yes | 24 | 100 | 5 | 7 | 70 |
| P09 | 69 | Men | Retired | Lives with others | Yes | 147,6 | 100 | 5 | 7 | 81 |
| P10 | 73 | Men | Retired | Lives with others | No | 99,6 | 100 | 4 | 8 | 73 |
| P11 | 73 | Men | Retired | Lives with others | No | 42 | 100 | 5 | 7 | 77 |
| P12 | 51 | Men | Disability pension | Alone | Yes | 36 | 95 | 5 | 10 | 79 |
| P13 | 72 | Men | Retired | Lives with others | No | 8,4 | 100 | 5 | 6 | 67 |
| P14 | 64 | Men | Disability pension | Lives with others | Yes | 49,2 | 85 | 4 | 4 | 77 |
| P15 | 65 | Men | Sick leave | Lives with others | No | 9,6 | 85 | 4 | 8 | 70 |
| P16 | 58 | Men | Disability pension | Lives with others | Yes | 30 | 80 | 5 | 3 | 45 |
| P17 | 71 | Men | Retired | Lives with others | No | 45,6 | 95 | 3 | 7 | 84 |
| P18 | 66 | Men | Disability pension | Lives with others | No | 68,4 | 100 | 5 | 6 | 78 |
| P19 | 59 | Men | Disability pension | Lives with others | Yes | 44,4 | 95 | 5 | 8 | 68 |
| P20 | 58 | Men | Sick leave | Alone | No | 7,2 | 95 | 5 | 8 | 67 |
| P21 | 61 | Woman | Disability pension | Lives with others | No | 147,6 | 100 | 5 | 9 | 75 |
| P22 | 45 | Woman | Disability pension | Lives with others | No | 129,6 | 100 | 5 | 8 | 72 |
| P23 | 58 | Men | Retired | Lives with others | No | 12 | 100 | 5 | 8 | 45 |
| P24 | 24 | Men | Disability pension | Lives with others | Yes | 36 | 95 | 5 | 6 | 71 |
| P25 | 62 | Men | Retired | Lives with others | No | 64,8 | 95 | 5 | 5 | 72 |
| P26 | 43 | Men | Disability pension | Lives with others | No | 79,2 | 100 | 5 | 10 | 76 |
| P27 | 27 | Woman | Disability pension | Lives with others | No | 103,2 | 100 | 5 | 8 | 54 |
| P28 | 52 | Men | Disability pension | Lives with others | No | 55,2 | 95 | 5 | 9 | 80 |
| P29 | 79 | Woman | Retired | Alone | Yes | 34,8 | 80 | 5 | 3 | 54 |
| P30 | 60 | Woman | Retired | Lives with others | Yes | 16,8 | 70 | 5 | 6 | 70 |
| P31 | 50 | Woman | Unemployed | Lives with others | No | 34,8 | 100 | 5 | 7 | 70 |
| P32 | 60 | Men | Disability pension | Lives with others | No | 39,6 | 90 | 4 | 9 | 65 |
| P33 | 46 | Men | Disability pension | Lives with others | Yes | 44,4 | 85 | 5 | 12 | 90 |
| P34 | 43 | Men | Employment | Lives with others | No | 45,6 | 100 | 5 | 8 | 84 |
| P35 | 70 | Men | Retired | Lives with others | Yes | 172,8 | 95 | 5 | 2 | 61 |
