# Supplementary material for: Lived Experiences of Returning to Participation After Mild Stroke: A Phenomenological Study in Spain
Source: Health Expect. 2026 Feb 24;29(2):e70573. doi: 10.1111/hex.70573 (PMC12932911; doi:10.1111/hex.70573)

**Supplementary material III.** Themes, groups of common meaning and meaning units that emerged from the participants' narratives.


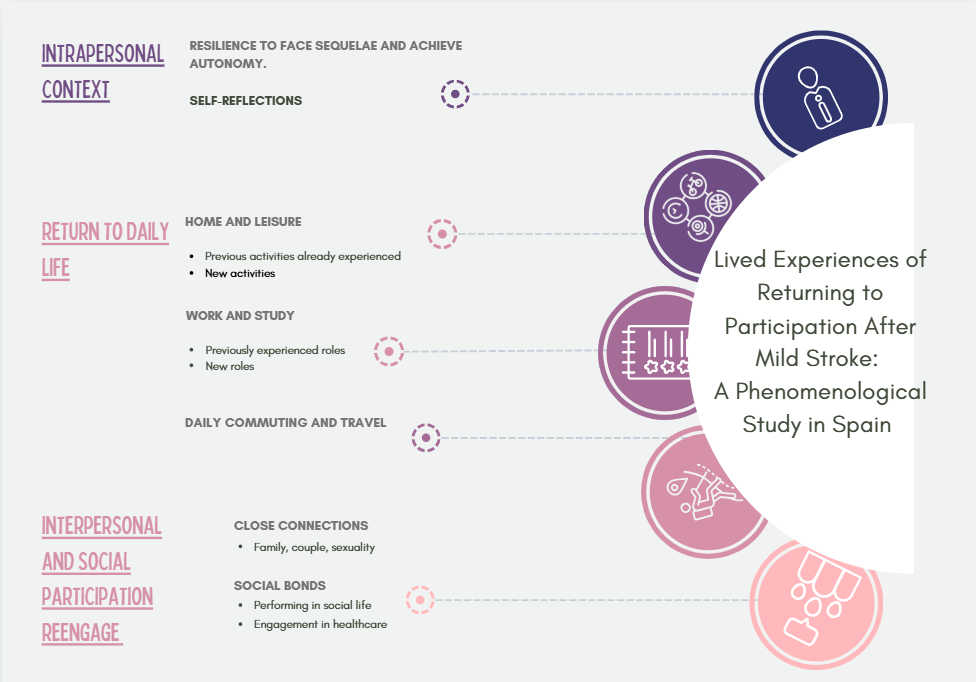

Supplement: Supplementary file 3 — Supplementary Material III: Themes, groups of common meaning and meaning units that emerged from the participants' narratives. [file HEX-29-e70573-s002.docx]
