# Supplementary material for: Lived Experiences of Returning to Participation After Mild Stroke: A Phenomenological Study in Spain
Source: Health Expect. 2026 Feb 24;29(2):e70573. doi: 10.1111/hex.70573 (PMC12932911; doi:10.1111/hex.70573)
Supplement: Supplementary file 4 — Supplementary Material IV: Additional supporting quotes for each theme. [file HEX-29-e70573-s003.docx]

**Supplementary Material IV:** Additional supporting quotes for each theme.

| **Intrapersonal context** |
| --- |
| *Resilience to face sequelae and achieve autonomy****.*** |
| *"It was a drama, not only because of the temperature, but also the sensation of the water falling on you. My muscles would begin to jerk. Taking a shower was, it was a real drama." (P23)*  *"I was at home and I didn't feel at home, because of course, it was a new house, ...in my kitchen I didn't have things where I could reach them... I had to readapt everything... I had to adapt the house a little bit to suit myself, because between what happened (the stroke), the separation and everything, well... "Where do I have...". So, then I started to put the kitchen where I see that I can get to, right? Doing everything my own way." (P02)*  *"In other words: "Well, don't worry, you'll see, we'll get better". That boosts my morale. But: "Well, that's no big deal". Well, that brings me down." (P29)*  *"…They helped you, but I struggled. If I could manage to button half a button today, the next day I could get three quarters in. The next day - the day you could get the whole button in - and successfully achieve it, was an enormous feat." (P15)* |
| *Self-reflections* |
| *"(This situation) I have taken it as a fuckup, my apologies... But well... You have to put up with it (he gets discreetly emotional, swallows saliva). I tell you, my life got put on a standstill, because everyone I know says: "It’s non-stop" ... and now I have to stop". (P06)*  *"I used to work a lot and I realized that having a disability I could live and I could do things that I liked; things I didn't have time to do before; so it was like a freedom." (P01)*  *"I would definitely say that a stroke is something tremendously, very, very, very... I don't dare to say 'horrible,' but it is a major cause of death. In women, it is the leading cause, and in men, well, I believe it's the third. But above all, from my own experience, I would like to convey the fragility of all people because, from one moment to the next, something like this can turn our lives upside down. However, at the same time, I want to emphasize that this doesn’t mean life is over—there is life after a stroke." (P10)*  *"You can't make plans or foresee what will happen; it just happens, and you have to accept it as it is. Simple as that, inevitable as that." (P21)*  *"I'm a very restless person; I always need to be doing something. But now, things look different. You rethink it and say to yourself, 'Life is short—how do I want to live it?'" (P31)* |
| **Return to daily life** |
| *Home and Leisure* |
| *"For example, the issue of ironing. It's a bit complicated because, of course, I have to use one hand to lay out the clothes in a special way ... and with the other hand I have to iron them, or look for some tweezers to hook the clothes, so that they stay taut." (P12)*  *"I manage almost everything in the house; the children help me a little bit, but I, come on, I'm always the one who manages everything and so on... And... I don't know, that, that makes me, that makes me feel good; to see that I do everything myself, that well, the children also do the cleaning and so on, but the, the food and everything and if I have to tell them something: "Do this to me". I have to tell them, because if not, they don't do it, right? That makes me feel good, of course. "(P 21)*  *Let's see, yesterday I thought of asking my wife: "Have you done the dishes? And I... I was there, with the fight, with the pot, staying there until I managed to wash it" (P 16).*  *"I used to play the guitar a lot; I’ve been playing the guitar all my life, but now I’m gradually forcing myself a little bit, especially because of therapy with the fingers of my left hand." (P23)*  *"I have to recover archery, because it’s something that gives me a lot of independence, pleasure, ...it makes me feel very capable." (P19)*  *"I used to fall in love with traveling, even if I was tired…Now I enjoy getting tired, which is different.". (P01)*    *"I've always liked reading and listening to music, but after the stroke I didn't feel like it... but I've been enjoying books and music for the last year or so." (P10) "In that regard, you have a lot more time to read. I read a lot. Yesterday I read half a book, (laughs). Or studying English, for example... I tried to do that before the stroke but I didn't have time and now I have" (P04)*    *"I have signed up for 3 or 4 outings with them and the truth is that, things I didn't do before, such as going to the mountains, now they take me in a wheelchair, eh?" (P20)*  *"Well, before the stroke I was very streetwise (…). After the stroke, my activity is at home, in the neighbourhood. Going out downtown is very difficult for me, because it is when there are lots of people... I am afraid that someone will push me and I will fall on the ground." (P13)*  *"Something I’ve discovered, which I mentioned to you before, is crafts. I've gotten into them, and I really enjoy it. I make flowers, miniatures, I paint boxes… I create… I don’t know, just that. Well, also knitting, crochet… A bit of everything." (P05)* |
| *Work and study* |
| *"Let's see, I was a professor at ESADE and I've done the work to be able to go back to being a professor at ESADE. Not with the same dedication as before, but it's all right. The thing is that "work" ... I wouldn't say "work", but I would say academic activity."(P18)*  *"So, of course, in my case, I only need an Internet connection and a computer. That's it. So, of course, that's very easy. Then I started to do a job, as I was saying, ehh very informal, that is, I offer my services to local businesses, so I have done a lot of web pages, applications... Well, all kinds of things and without being professional, because I can't, I can't meet deadlines."(P26)*  *"At the beginning, the first few times I was put in as a senior resident so I wouldn't be alone in the practice."(P34).*  *"I actually fought a lot when I was training, because I wanted to learn, which was minor surgery. And I don't do that now. Because of course, I know that my hand for suturing is not there and I have done that many times and it makes me very angry not to be able to do it." "(P34)*  *"I have been doing this housework for the last 6 months, and the housework does not specify that it is daily, it is daily from Monday to Sunday, exactly because on Sunday you wash smooth iron, that is, this is much more than work (P 01).*  *"I live my life the same as before. Well, work, I used to work and now I don't. That's what I miss: working." (P25)*  *"I thought I would be able to work. Work two hours, three hours, but that’s not the case. Not right now... And in the end, I accepted it, because... I realised that I couldn't." (P19)*  *"… the cognitive, intellectual activity, that's great and I have a lot of fun with that. So of course, since I'm not allowed to work, it sounds very cool, but it's not as cool as it sounds because I used to love work." (P26)*    *"It's part of my rehabilitation, I mean, for example, I know that before my stroke, a web page took me a weekend... nowadays, it might take me a month." (P26)*  *"It's just that the feeling, ... that if I go back to the day-to-duties. it's like the feeling that everything is, everything is taken care of. It's like a complete return to normality." (P34)*  *"I put my role as a student on hold because of online classes. At first, I struggled to concentrate and couldn’t keep up with the teacher. But I’ll pick it up again in September, and I hope it goes well for me." (P13)*  *"Before the stroke, I was pretty lazy… but now, I see things differently. I wouldn’t call it a necessity, because I do enjoy it, but I also see it as my new job, something I have to do. I realize how much I’ve progressed, and I don’t want to stop—just to stay comfortable and take it easy. I don’t want to lose what I’ve gained; I want to keep moving forward as much as possible." (P15)* |
| *Daily Commuting and Travel* |
| *"Driving also marked me a lot because I liked driving very much and I was very independent and that really made an impression on me, it makes me really angry every time I have to be driven." (P34)*    *"Before it was terrible for me, because maybe I didn't know how to tell the driver where I was going. Now, increasingly less so, because I realized that I can easily talk to the coach driver and tell him." (P21)*  *"Driving has been wonderful. I'm still very scared, but it's been wonderful." (P12)*  *"The day we had to go to, to visit, to visit the puppies we adopted, ...well we went in my car and it was wonderful. So, (clicks tongue) let's see, the recovery is not complete - it is still going on - and the, the top will come, ...but I still have improvement potential." (P18)*  *"It’s something that´s very, that´s very fundamental for me. It's to be able to access with my car to the orchard, you know, because it's far away. It's, it's, it's - just to give you an idea - 6, 6 kilometers from, from, from, from where I live. By the way, I know that now I definitely need - I need a car, you know? So - since I have a car and I can - and I can drive - well, I think it's - it's great for me." (P 17)*  *"The car serves me to have an independence that, I didn't think I was going to have."(P32)*  *"I get in the car and I go shopping and I have a great deal of autonomy and... I'm back to being more like a pre-ictus."(P33)*  *"I already move in a more or less social environment and well, at the end sometimes I take the car and I go to the Costa Brava for an hour, an hour and a half driving."(P09)* |
| **Interpersonal and Social Participation** |
| *Close Connections* |
| *"Of course, at the beginning it was an odyssey to tie a boot, tying the lace was science fiction, and my wife said, "Well, should I buy you something with Velcro? And I said no." (P23)*  *"To be clear, I can't do... I can't have relations as I had them before... because of positions and this kind of thing... at the beginning, it was very difficult for me. Now I have problems and in addition ..., I am on an antidepressant that affects me sexually." (P19)*  *"We have commented many times with my wife. Well, listen, I would have liked to take the two granddaughters we have to school... I would have liked to, but..., the older one may be possible, but the younger one, we have to take her safely and I don't dare." (P10)*  *"Let’s say they give me some room to push myself as much as I can. When they see I’ve reached my limit, my husband steps in, and we talk about household things and all that. We talk pretty much like we used to." (P07)*  *"I've regained the experience of being in a relationship, which I didn’t have before. But now I do—I've been with my partner for over a year now. Right now, they’re here on vacation, spending time with me."(P12)* |
| *Social Bonds* |
| *"I don't know how to put it. I'm more distant from everything, although, we talked before about so much social network and that I have so many friends, there are also a lot of things that, at the level of communication, I don't know how to do..." (P08)*  *"I have also resumed my English classes. Now that the pandemic is over and the good weather has returned, I have signed up for English classes again. I go there with people I have known since my stroke. It's a group of friends who meet to study English... and to talk a little." (P18)*  *"I started to make more friends after the stroke than before. I met a lot of people." (P27)*  *"When I had my stroke, I was taking care of my mother, who had also had a stroke. And then, six months later, it happened to me. So now, my siblings take care of my mother, and my friends are the ones who help me." (P06)*  *"I don’t take advantage of my friends either because everyone tells me, --Miguel, whenever you need to go shopping, call me 'I’ll go with you when you need to carry things'.” (P08)* |
| *Engagement in Healthcare* |
| *"Well, here (at the association), it is better because they are more individualistic, they take better care of you and they are more attentive to you. There, in the hospital there are so many (patients) and of course, they can't, they know they are going to be with you for three months." (P25)*  *"Just having a moment where people actually explain things to you… Especially people at the association, who tell you a little about what has happened to you, what’s going on. Having someone explain it instead of just saying, 'You're fine, don’t worry.' Because they hand you a paper full of words you don’t understand and say, 'Here, you’re good now. Go home. Here’s your report.'” (P20)*  *"The physical therapist—well, at the stroke association, occupational therapy taught me how to manage with my good hand. How to get things done and use my mind to figure them out. That really helped me move around and be able to do things." (P25)*  *"The head of physiotherapy, really nice, gave me a lot of explanations from the beginning. Since I was the youngest there by 30 years—imagine that—maybe that’s why they informed me more. I asked a lot of questions, and more than as part of the treatment, it was because of the relationship we had. They didn’t give me a ‘masterclass,’ but they did explain all the effects very well." (P17)* |
